# Effects of supervised exercise training on lower-limb cutaneous microvascular reactivity in adults with venous ulcers

**DOI:** 10.1007/s00421-017-3772-0

**Published:** 2017-12-02

**Authors:** Garry A. Tew, Anil Gumber, Emma McIntosh, Sue Kesterton, Brenda King, Jonathan A. Michaels, Markos Klonizakis

**Affiliations:** 1 Department of Sport, Exercise and Rehabilitation, Northumbria University, Newcastle-upon-Tyne, NE1 8ST UK; 2 Centre for Health and Social Care Research, Sheffield Hallam University, Sheffield, S10 2BP UK; 3 Centre for Sport and Exercise Science, Sheffield Hallam University, Sheffield, S10 2BP UK; 4 Manor Clinic, Sheffield Teaching Hospitals NHS Foundation Trust, Sheffield, S12 2ST UK; 5 School of Health and Related Research, University of Sheffield, Sheffield, S1 4DA UK

**Keywords:** Randomized controlled trial, Exercise, Ulceration, Vascular function, Laser Doppler fluxmetry, Iontophoresis

## Abstract

**Purpose:**

To investigate the effects of a 12-week supervised exercise programme on lower-limb cutaneous microvascular reactivity in adults with venous leg ulceration.

**Methods:**

Thirty-eight adults with unilateral venous ulceration who were being treated with lower-limb compression therapy (58% male; mean age 65 years; median ulcer size 5 cm^2^) were randomly allocated to exercise or control groups. Exercise participants (*n* = 18) were invited to attend thrice weekly sessions of lower-limb aerobic and resistance exercise for 12 weeks. Cutaneous microvascular reactivity was assessed in the gaiter region of ulcerated and non-ulcerated legs at baseline and 3 months using laser Doppler fluxmetry coupled with iontophoresis of acetylcholine (ACh) and sodium nitroprusside (SNP). Cutaneous vascular conductance (CVC) was calculated as laser Doppler flux (AU)/mean arterial pressure (mmHg).

**Results:**

Thirty-seven participants completed follow-up assessments. Median session attendance was 36 (range 2–36). Analyses of covariance revealed greater peak CVC responses to ACh in the exercise group at 3 months in both the ulcerated (adjusted difference = 0.944 AU/mmHg; 95% CI 0.504–1.384) and non-ulcerated (adjusted difference = 0.596 AU/mmHg; 95% CI 0.028–1.164) legs. Peak CVC responses to SNP were also greater in the exercise group at 3 months in the ulcerated leg (adjusted difference = 0.882 AU/mmHg; 95% CI 0.274–1.491), but not the non-ulcerated leg (adjusted difference = 0.392 AU/mmHg; 95% CI − 0.377 to 1.161).

**Conclusion:**

Supervised exercise training improves lower-limb cutaneous microvascular reactivity in adults with venous leg ulceration.

## Introduction

Venous ulcers are the most common type of leg ulcers, accounting for approximately 70% of cases (Agale 2013). The prevalence of venous ulceration increases with age, with 1–3% of the population aged over 60 years being affected (Graham et al. 2003). Venous valve incompetence and calf muscle pump dysfunction can result in retrograde blood flow and venous hypertension (Meissner et al. 2007). The pathogenic steps leading from venous hypertension to ulceration have not been fully elucidated; however, the close relationship between capillary rarefrction, cutaneous hypoxia, a lack of cutaneous vascular reserve, and the clinical progression of trophic skin changes (Jünger et al. 2000) suggest that cutaneous microangiopathy may be implicated.

Compression therapy, delivered using compression hosiery or multi-layer bandaging, is the mainstay of treatments for venous leg ulcers (Scottish Intercollegiate Guidelines Network 2010). The objective is to provide graded external compression to the leg and oppose the hydrostatic forces of venous hypertension (Eberhardt and Raffetto 2014). Lifestyle factors, including exercise, nutrition, and smoking, are also mentioned in guidelines on the management of venous ulceration and chronic venous insufficiency (Scottish Intercollegiate Guidelines Network 2010); however, these factors receive relatively little emphasis. Exercise training that targets improvements in calf muscle pump function, ankle range of motion and cutaneous microvascular function might be a potentially useful adjunct therapy for enhancing ulcer healing, given that abnormalities in these factors are associated with prolonged healing (Jünger et al. 2000; Barwell et al. 2001; Simka 2007). Previous work has shown that lower-limb resistance training can improve calf muscle pump function, ankle range of motion and lower-limb haemodynamics in people with venous ulceration (Kan and Delis 2001; Jull et al. 2009; O’Brien et al. 2013). Our group has also demonstrated that lower-limb endurance training (Klonizakis et al. 2009), but not upper-limb endurance training (Klonizakis et al. 2010), can improve lower-limb cutaneous microvascular reactivity in people who had recently had surgery for varicose veins. However, a recent systematic review concluded that further research is required to determine whether exercise training has a beneficial effect on ulcer healing and health-related quality of life (Yim et al. 2015).

In light of this background evidence, we hypothesised that a specially designed supervised exercise programme delivered in conjunction with compression therapy would be a more effective means of promoting venous ulcer healing than compression therapy alone. Before embarking on an appropriately powered randomised trial to test this hypothesis, we have undertaken a smaller-scale feasibility trial to address areas of uncertainty (e.g., the ability to recruit and retain participants) and to investigate the effects of our exercise programme on lower-limb cutaneous microvascular reactivity. Herein, we report the microvascular data; the feasibility and acceptability aspects have been published elsewhere (Klonizakis et al. 2017).

## Methods

A full description of the protocol for this randomised controlled trial is available elsewhere (Tew et al. 2015). Thirty-eight adults who were receiving lower-limb compression for a new venous leg ulcer of greater than 1 cm diameter were recruited from tissue viability clinics and newspaper advertisement in Sheffield, United Kingdom. Patients were excluded if they: had an ankle-brachial pressure index below 0.80; were unsuitable to exercise; were pregnant, awaiting major surgery, or had insulin-dependent diabetes mellitus, or if they had experienced an ulcer at the same site within the previous 3 months. Ethics approval was granted by the NHS National Research Ethics Service, Yorkshire and the Humber (Sheffield) Committee (14/YH/0091), and all participants provided written informed consent prior to enrolment. The trial was prospectively registered (Current Controlled Trials ISRCTN09433624).

### Randomisation and interventions

Following baseline assessments, participants were randomly assigned to receive usual care (*n* = 20) or usual care plus a 12-week supervised exercise programme (*n* = 18). The randomisation was stratified by ulcer size (maximum ulcer diameter of either between 1 and 3 cm or greater than 3 cm in any direction) with permuted blocks of variable size, because this variable is a known predictor of ulcer healing (Margolis et al. 1999).

Usual care comprised of individualised lower-limb compression therapy (provision of multi-layer bandages or two-layer stockings), standard advice to elevate the affected leg, and tissue viability clinic appointments as often as was deemed clinically necessary. Participants allocated to the exercise group were invited to attend three 60-min sessions of supervised aerobic and resistance training each week for 12 weeks (total of 36 sessions) at Sheffield Hallam University. Each session began with 5 min of low-intensity treadmill walking or cycling for a warm-up. The subsequent aerobic component involved a mixture of incline treadmill walking and upright cycle ergometry for 30 min at a perceived exertion of 12–14 (i.e., somewhat hard) on Borg’s 6–20 scale (Borg 1982). The use of relative intensities helped ensure that the exercise programme had inherent progression as the participants became fitter. Participants then completed two to three sets of four lower-body resistance exercises (e.g., body-weight squats, calf raises, and bench step-ups). For each set, a resistance (e.g., hand-held dumbbell) was selected so the participant experienced moderate muscle fatigue within 10–15 repetitions. Each session ended with a 5-min cooldown of low-intensity walking or cycling, followed by static stretches for all of the major muscle groups of the legs (3 × 20-s stretch per muscle group held at the point of mild discomfort).

### Assessment of lower-limb cutaneous microvascular reactivity

Cutaneous microvascular reactivity was assessed in the gaiter region of both legs at baseline and 3 months using laser Doppler fluxmetry coupled with incremental-dose iontophoretic administration of acetylcholine (ACh) and sodium nitroprusside (SNP). All microvascular assessments were performed in a temperature-controlled room (range 22–24 °C) following an acclimatisation period of at least 15 min. During assessments, participants lay supine with the legs elevated at 30°. Participants were asked to refrain from exercise and consuming caffeine or alcohol for 24 h before study assessment visits.

The gaiter area of the leg to be studied (ulcerated leg first, non-ulcerated leg second; 10 min passive break between legs) was cleaned with an alcohol wipe and allowed to dry before two drug delivery electrodes (PF383; Perimed AB, Jarfalla, Sweden) were applied to the skin surface. These electrodes were positioned approximately 4 cm apart over healthy-looking skin, typically 4–8 cm proximal to the medial malleolus and at least 3 cm from the edge of the ulcer (for the ulcerated limb). One of the electrodes contained 80 μl of the endothelium-dependent vasodilator, ACh (Miochol-E, Novartis, Stein, Switzerland), and the other 80 μl of the endothelium-independent vasodilator, SNP (Nitroprussiat; Rottapharm, Monza, Italy). Drug concentrations of 1% were used with deionised water as the solvent.

A battery-powered iontophoresis controller (PeriIont PF382b; Perimed AB) was used to provide the charge needed for delivering ACh and SNP across the skin barrier. The anodal (positive) current was used to transfer ACh, with the cathodal (negative) current used to transfer SNP. A 4-min recording of baseline flux was followed by administration of the two agents according to the following protocol: 0.2 mA for 10 s (2 mC), 0.2 mA for 15 s (3 mC), 0.2 mA for 20 s (4 mC), and 0.3 mA for 20 s (6 mC), with a 4-min recording period between each dose (Klonizakis et al. 2009). This protocol was chosen as it is sufficient to provide effective ACh and SNP delivery, while largely avoiding the non-specific vasodilation observed with higher electrical charges (Droog et al. 2004).

To obtain an index of skin blood flow, cutaneous red cell flux was measured by placing an iontophoresis laser Doppler probe (PF481-1; Perimed AB), connected to a laser Doppler fluxmeter (PF5001; Perimed AB), in the centre of each drug delivery electrode. The laser Doppler probe signals were continuously monitored via an online software chart recorder (PSW; Perimed AB). Heart rate (Sports Tester; Polar, Kempele, Finland) and blood pressure (left arm; Dinamap Dash 2500; GE Healthcare, Wauwatosa, WI, USA) was monitored at 5-min intervals throughout the protocol.

Measurements of red cell flux (recorded in arbitrary units, AU) were divided by adjusted mean arterial pressure values (in mmHg) to give cutaneous vascular conductance (CVC) in AU/mmHg. Adjustment of mean arterial pressure was done to account for the effects of the legs being elevated at 30°, was based on the data of Nielsen (1983), and simply involved subtracting 24.259 from each mean arterial pressure value before calculating CVC. The peak CVC responses to ACh and SNP (typically observed after the 6-mC charge) were used as indicators of microvascular endothelial-dependent and -independent vasodilation, respectively. The technical error of measurement for drug-induced peak flux responses in our laboratory is 15%.

### Statistical analysis

The effect of the exercise programme on cutaneous microvascular reactivity was evaluated using analysis of covariance (ANCOVA) models. The 3-month outcome was the dependent variable and trial arm (exercise and control) was the independent variable. The baseline value of the outcome was included as a covariate (Vickers and Altman 2001). Mann–Whitney tests of the 3-month outcome were used when data were non-normally distributed and could not be transformed to conform to normality. The analyses were done on an intention-to-treat basis, including only those participants with both baseline and follow-up data available (i.e., complete case analysis). The treatment effect (exercise minus control) is presented with its 95% confidence interval (CI) and *P* value. Statistical significance was set at *P* ≤ 0.05. The term “adjusted” is used to indicate when a value has been adjusted for the covariate (i.e., the baseline value). Analyses were conducted using IBM SPSS Statistics Version 22 (IBM United Kingdom Limited, Hampshire, United Kingdom).

## Results

Thirty-eight participants were randomised: 18 to exercise and 20 to control. Twenty-two (58%) of the participants were male and the mean age was 65 years (range 37–81). The median ulcer size and duration of ulcer at baseline was 5.1 cm^2^ (range 1.3–136.4 cm^2^) and 5 months (range 0.5–72 months), respectively. The most common comorbidities were hypertension (*n* = 11) and non-insulin dependent type 2 diabetes (*n* = 8). The participants in the two groups had similar baseline characteristics (Table 1), although there was a greater percentage of males in the control group (70 versus 44%).

**Table 1:** Participant characteristics

Variable	Exercise group (*n* = 18)	Control group (*n* = 20)
Age (years)	66.9 (13.9)	62.3 (10.8)
Sex, number male/female	8/10	14/6
Stature (cm)	171.1 (11.9)	171.1 (10.7)
Body mass (kg)	102.1 (29.4)	106.0 (24.4)
Ulcer size (cm^2^), median (range)	4.9 (1.9–136.4)	5.4 (1.3–56.6)
Duration of ulcer, months, median (range)	5 (1–72)	5 (0.5–36)
Ankle-brachial index^a^	1.05 (0.14)	1.09 (0.18)
Ankle circumference^a^ (cm)	27.1 (5.5)	26.7 (4.8)
Calf circumference^a^ (cm)	37.3 (7.6)	41.8 (7.3)
Comorbidities, *n* (%)		
Hypertension	7 (39)	4 (20)
History of other CVD	1 (6)	7 (35)
Non-insulin dependent diabetes	4 (22)	4 (20)
History of cancer	2 (11)	1 (5)
Hypercholesterolemia	1 (6)	2 (10)
Medications, *n* (%)		
Anti-platelet/anti-coagulant	7 (39)	5 (25)
Statin	3 (17)	5 (25)
ACE-inhibitor	1 (6)	1 (5)
Beta-blocker	3 (17)	5 (25)
Calcium channel blocker	1 (6)	2 (10)
Diuretic	4 (22)	3 (15)

Values are mean and SD unless otherwise stated

*ACE* angiotensin converting enzyme, *CVD* cardiovascular disease

^a^Ulcerated leg

### Participant retention and intervention attendance

One person withdrew from the study before completing the 3-month assessments. This person, who was in the exercise group, withdrew after having completed four exercise sessions due to having an infected ulcer. The infection was deemed unrelated to the study. A further four participants did not complete the exercise programme due to unrelated health reasons, but did complete the follow-up assessments. These participants had completed 2, 6, 15 and 17 exercise sessions, respectively, before leaving the exercise programme. The remaining 13 participants all had 100% attendance records (36/36 sessions). Overall, 512 out of a possible 648 sessions were attended (79%), with median session attendance being 36 (range 2–36).

### Differences in cutaneous microvascular reactivity between ulcerated and non-ulcerated legs at baseline

Table 2 shows the unadjusted peak CVC responses to ACh and SNP at baseline and 3 months, for both the ulcerated and non-ulcerated legs. At baseline, peak CVC responses to both ACh and SNP were greater in the non-ulcerated leg compared with the ulcerated leg: mean differences = 0.508 AU/mmHg (95% CI 0.218–0.797; *P* = 0.001) and 0.507 AU/mmHg (0.314–0.700; *P* < 0.001), respectively (analysis based on pooled data from both groups).

**Table 2:** Peak cutaneous vascular conductance (CVC; measured in AU/mmHg) responses to acetylcholine (ACh) and sodium nitroprusside (SNP)

Variable	Exercise (*n* = 17)		Control (*n* = 20)	
	Baseline	3 months	Baseline	3 months
Ulcerated leg				
ACh—peak CVC	0.684 (0.310)	1.674 (0.874)	0.774 (0.266)	0.728 (0.344)
SNP—peak CVC	0.709 (0.384)	1.796 (1.234)	0.686 (0.353)	0.917 (0.442)
Non-ulcerated leg				
ACh—peak CVC	1.328 (1.257)	1.875 (1.047)	1.174 (0.556)	1.248 (0.648)
SNP—peak CVC	1.229 (0.977)	1.858 (1.399)	1.198 (0.666)	1.462 (0.854)

Values are unadjusted mean and SD

### Effects of 12-week exercise training on cutaneous microvascular reactivity in ulcerated and non-ulcerated legs

**Fig. 1:**
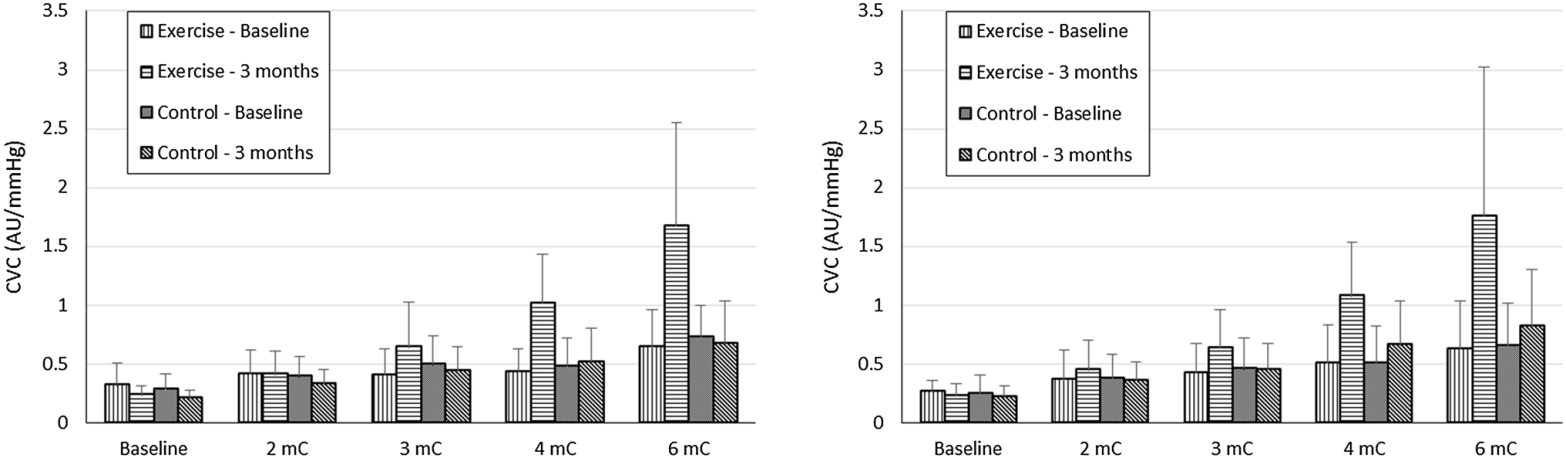
Cutaneous vascular conductance (CVC) responses at each stage of acetylcholine (ACh; left panel) and sodium nitroprusside (SNP; right panel) administration on the ulcerated leg. Values are unadjusted mean and SD

**Fig. 2:**
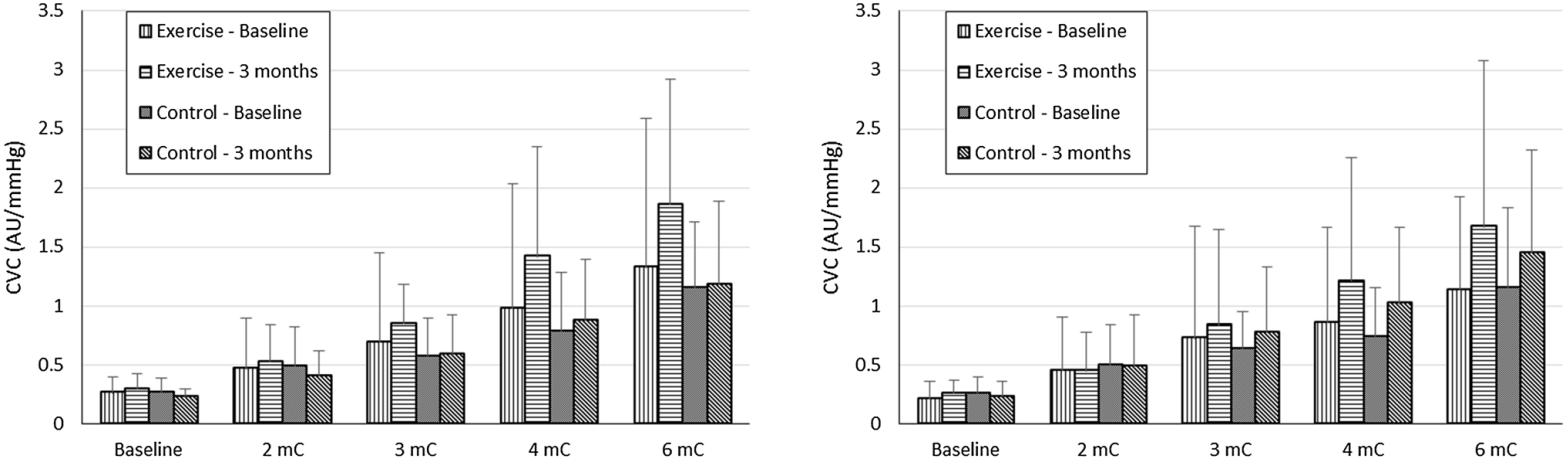
Cutaneous vascular conductance (CVC) responses at each stage of acetylcholine (ACh; left panel) and sodium nitroprusside (SNP; right panel) administration on the non-ulcerated leg. Values are unadjusted mean and SD

For the ulcerated leg, the adjusted peak CVC responses to both ACh and SNP were greater in the exercise group at 3 months: mean differences = 0.944 AU/mmHg (95% CI 0.504–1.384; *P* < 0.001) and 0.882 AU/mmHg (0.274–1.491; *P* = 0.006), respectively. Unadjusted data for the ulcerated leg are shown in Fig. 1. The adjusted peak CVC response to ACh was also greater in the exercise group for the non-ulcerated leg at 3 months: mean difference = 0.596 AU/mmHg (95% CI 0.028–1.164; *P* = 0.04). The adjusted peak CVC response to SNP was on average 0.392 AU/mmHg greater in the exercise group at 3 months (95% CI − 0.377 to 1.161); however, this was not statistically significant (*P* = 0.307). Unadjusted data for the non-ulcerated leg are shown in Fig. 2. The ANCOVA assumption of homogeneity of variances was violated in both the ACh and SNP analyses for the non-ulcerated leg. As this violation could not be resolved using log-transformation, we conducted Mann–Whitney *U* tests to compare the 3-month follow-up data between groups. The *P* values were consistent with the results of the ANCOVA analyses: peak ACh, *P* = 0.042; peak SNP, *P* = 0.537.

### Differences in cutaneous microvascular reactivity between ulcerated and non-ulcerated legs at 3 months

At 3 months, the peak CVC responses to ACh and SNP continued to be greater in the non-ulcerated leg compared with the ulcerated leg in the control group [*n* = 20; ACh—peak CVC: 1.248 (SD 0.648) vs. 0.728 (SD 0.344) AU/mmHg, *P* = 0.001; SNP—peak CVC: 1.462 (SD 0.854) vs. 0.917 (SD 0.442) AU/mmHg, *P* = 0.001], but not the exercise group [*n* = 17; ACh—peak CVC: 1.875 (SD 1.047) vs. 1.674 (SD 0.874) AU/mmHg, *P* = 0.347; SNP—peak CVC: 1.858 (SD 1.339) vs. 1.796 (SD 1.234) AU/mmHg, *P* = 0.875] (‘ulcerated leg’ = leg that had the ulcer at baseline, regardless of whether or not the ulcer had healed at 3 months).

Median ulcer size at 3 months across both groups was 1.5 cm^2^ (range 0–78.5 cm^2^). At this time point, complete ulcer healing was apparent in nine of the 17 (53%) exercise participants and three of the 20 (15%) control participants. Peak CVC responses were greater in healed legs (*n* = 12) compared with non-healed legs (*n* = 25) for both ACh [1.668 (SD 1.000) vs. 0.920 (SD 0.547) AU/mmHg, *P* = 0.006] and SNP [1.995 (SD 1.332) vs. 0.997 (SD 0.563) AU/mmHg, *P* = 0.003].

## Discussion

The present study investigated the effects of a 12-week programme of lower-limb aerobic and resistance exercise on lower-limb cutaneous microvascular reactivity in adults with unilateral venous ulceration. The principal findings are as follows: (1) at baseline, the peak CVC responses to the endothelium-dependent vasodilator ACh and the endothelium-independent vasodilator SNP were depressed in ulcerated legs compared with non-ulcerated legs; (2) 12 weeks of exercise training improved the peak CVC responses to both ACh and SNP in the ulcerated leg, and to ACh (but not SNP) in the non-ulcerated leg; (3) the between-limb differences in cutaneous microvascular reactivity that were observed at baseline were no longer apparent in the exercise group at 3 months, and; (4) peak CVC responses to both ACh and SNP were greater in healed versus non-healed legs at 3 months.

The microvascular reactivity data were obtained using the reproducible and validated method of iontophoresis to deliver ACh and SNP to the skin microvasculature in a controlled manner without trauma, and laser Doppler fluxmetry to assess microvascular erythrocyte flux (Morris and Shore 1996; Berghoff et al. 2002; Tew et al. 2010). Although the galvanic effect of current and voltage application with iontophoresis can cause non-specific vasodilation (Berghoff et al. 2002), the use of 1% drug concentrations and currents ≤ 0.3 mA should have minimised such effects (Droog et al. 2004). In addition, as the protocol was consistent between groups before and after the intervention period, it is unlikely that any observed between-limb differences or changes in microvascular responses are due to non-specific, protocol-related vasodilatory effects.

The CVC response to ACh was used to assess microvascular endothelial function in vivo, whereas the CVC response to SNP was used to assess microvascular smooth muscle function (Morris and Shore 1996). ACh interacts with the M_3_ muscarinic receptor on the endothelial cell surface, which initiates a sequence of intracellular events—G protein activation, phospholipase A and/or C activation and stimulation of endothelial nitric oxide (NO) synthase activity—leading to NO synthesis, although prostacyclin and hyperpolarising factor release may also be induced (Berghoff et al. 2002; Holowatz et al. 2005; Kellogg et al. 2005; Fujii et al. 2013; Brunt et al. 2015). The NO diffuses across the endothelial cell membrane and basement membrane, binds to guanylate cyclase within the vascular smooth muscle cell, leading to an increase in intracellular cyclic guanosine monophosphate and, ultimately, smooth muscle relaxation and vasodilation. By contrast, SNP decomposes to release NO in vivo, which interacts with the vascular smooth muscle guanylate cyclase to produce vasodilation in an endothelium-independent manner (Turner et al. 2008).

The depressed vascular responses to ACh and SNP in ulcerated versus non-ulcerated legs (at baseline), therefore, indicate that aspects of cutaneous microvascular endothelial function and smooth muscle responsiveness to NO are disturbed in limbs with venous ulceration. Although the ACh and SNP data are consistent with that of previous work in earlier stage venous disease (Klonizakis et al. 2003), they contradict the findings of Ardron et al. (1991) who reported no significant differences in blood flux responses to ACh and SNP on the dorsum of the foot between 15 people with venous leg ulcers and 15 age and sex-matched healthy controls. The reasons underpinning these discrepant findings are unclear, but might be due to differences in study design; specifically in relation to the measurement site (gaiter region versus foot dorsum) and comparator (within-participant comparison of ulcerated and non-ulcerated legs versus between-participant comparison of patient and control feet). Other work by Jünger et al. (2000) has shown that cutaneous vascular reserve, as determined by the increase skin blood flow after a 3-min arterial occlusion (i.e., post-occlusive reactive hyperaemia), is exhausted at the site of a venous ulcer (healthy control group: + 721 ± 490%; in the ulcer: + 20 ± 41%, *P* < 0.01). Together, the available data suggest that lower-limb cutaneous microvascular reactivity is markedly impaired in people with advanced venous disease, at least in the gaiter region where most venous ulcers occur.

The mechanisms of cutaneous microvascular dysfunction in limbs with venous disease/ulceration remain unclear, although several possibilities exist. In venous disease, venous stasis in the microcirculation results in low shear stress on the endothelium, which leads to endothelial glycocalyx disruption and activation of endothelial cells and leucocytes by a variety of adhesion molecules and leucocyte chemoattractants (Raffetto 2016). This causes a decrease in endothelial NO synthase mRNA and protein expression that limits the bioavailability of NO (Qiu et al. 2013) and, potentially because of this, the vascular responsiveness to ACh. Alternatively, the impaired microvascular reactivity might be due to the localised persistent inflammatory state that is characteristic of venous ulcers (Raffetto 2016). Indeed, studies in rheumatoid arthritis have demonstrated that C-reactive protein (a blood marker of inflammation) correlates negatively with both endothelial-dependent and endothelial-independent microvascular function (Galarraga et al. 2008; Foster et al. 2010). Venous hypertension also causes microvascular structural abnormalities including a decrease in the number of capillaries and glomerulus-like changes in capillary morphology (Jünger et al. 2000), which might impact upon microvascular responsiveness to ACh and SNP. It should be noted that blood flow responses in the microcirculation assessed by laser Doppler are to a certain degree dependent on microvascular structure (Gardner-Medwin et al. 1997), so we cannot safely say that the between-limb differences in ACh and SNP responses are not influenced by vascular structural alterations. However, baseline (i.e., pre-stimulation) flux, which is a crude indicator of microvascular structure (Gardner-Medwin et al. 1997), was similar between ulcerated and non-ulcerated legs (Figs. 1, 2). It could also be argued that skin thickening due to lipodermatosclerosis seen in limbs with venous hypertension inhibited the iontophoretic delivery of ACh and SNP into the cutaneous circulation, thus reducing the amount of chemical available to initiate vasodilation. To minimise this possibility, we performed measurements away from the ulcer site, avoiding thickened skin or areas of oedema.

The principal novel finding of this study was that a 12-week supervised exercise programme improved measures of cutaneous microvascular reactivity in people being treated with compression therapy for venous ulceration. The data are broadly consistent with the results of a recent meta-analysis of seven controlled trials (*n* = 245), which showed that aerobic exercise training had a moderate and statistically significant beneficial effect (standardised mean difference = 0.43, 95% CI 0.08–0.78, *P* = 0.013) on cutaneous microvascular reactivity in a mixed population of adults who were primarily inactive at baseline (Lanting et al. 2017). Of the three trials that had used iontophoresis or microdialysis with ACh to assess cutaneous microvascular endothelial function (Klonizakis et al. 2009; Middlebrooke et al. 2006; Black et al. 2008), two reported a beneficial effect of training (Klonizakis et al. 2009; Black et al. 2008). Interestingly, our findings also demonstrated that a training effect was apparent for both ulcerated and non-ulcerated limbs, which may indicate both a therapeutic and preventative role of exercise training. The greater improvements observed in ulcerated legs might be due to a relatively depressed endothelial function at baseline (i.e., greater room for improvement).

The endothelial-dependent pathways and signalling events responsible for the improvement in ACh-induced vasodilation are largely unknown; however, an increase in NO bioavailability is a particularly plausible contributory factor given that aerobic exercise training (thrice weekly, 30 min at 30% heart rate reserve) has been shown to increase the NO-component of ACh-induced vasodilation in previously sedentary, healthy older adults (Black et al. 2008). Previous work indicates that improvements in NO-mediated vasodilator function might occur via the episodic increases in endothelial shear stress that occur with repeated bouts of exercise (Green et al. 2010). Shear stress upregulates endothelial NO synthase production in conduit arteries (Hambrecht et al. 2003), and Green et al. (2010) demonstrated that restricting increases in shear stress during an 8-week programme of repeated skin heating (bilateral forearm immersion in 42 °C water for 30 min, thrice weekly; an experimental model used to simulate exercise-induced thermoregulatory vasodilation) prevented improvements in conduit artery and cutaneous microvascular function from occurring. It is likely that the increases in cutaneous blood flow that occur acutely during exercise are due to a combination of thermoregulatory vasodilation and the direct action of the calf muscle pump (Eze et al. 1996; Simmons et al. 2011). Increasing core temperature and activating the calf muscle pump were therefore, important considerations during the design of the exercise programme. The focus on lower-limb exercise training was also informed by our previous studies showing that lower-limb endurance training (Klonizakis et al. 2009), but not upper-limb endurance training (Klonizakis et al. 2010), improved lower-limb cutaneous microvascular reactivity in post-surgical varicose vein patients.

That exercise training also improved the vascular responsiveness to SNP in ulcerated legs was a somewhat surprising finding given that several studies have shown no significant effect (Klonizakis et al. 2009; Tew et al. 2010; Wang 2005), including our aforementioned lower-limb exercise training study in post-surgical varicose vein patients (Klonizakis et al. 2009). However, Hodges et al. (2010) demonstrated that SNP-responsiveness was increased after 36 weeks of exercise training in post-menopausal women. Therefore, it may be that exercise training improves cutaneous vascular smooth muscle responsiveness to NO after longer periods of training (e.g., > 36 weeks), or that adaptations only occur in individuals with sub-optimal function at baseline. The latter might be more relevant to the present study given the shorter (12 week) programme duration, the fact that SNP responses were lower in ulcerated versus non-ulcerated legs at baseline, and that exercise training did not cause a significant improvement in SNP-responsiveness in non-ulcerated legs.

The role of impaired cutaneous microvascular reactivity in the pathogenesis of venous ulceration and the clinical significance of the exercise-induced improvements in cutaneous microvascular reactivity requires further research. For logistical reasons we were unable to perform additional interim microvascular assessments to explore if changes in cutaneous microvascular reactivity preceded ulcer healing. Although several factors are thought to influence susceptibility to ulceration and ulcer healing (Raffetto 2016), the functional integrity of the microcirculation to maintain blood flow, tissue oxygenation, and nutrient delivery is likely to be particularly important (Iabichella et al. 2006). This is supported by the work of Jünger et al. (2000), which indicated that various microvascular adaptations precede and determine ulcer healing. The generally large effect sizes and the fact that microvascular reactivity was superior in healed versus non-healed limbs at the 3-month assessment also support the notion that the observed changes may at least play some role in facilitating ulcer healing.

Strengths of this study include the randomised controlled study design, the low rates of attrition and missing data, the objective assessment of cutaneous microvascular reactivity, and the close adherence to the exercise and usual care interventions. Limitations include the relatively small sample size (which precluded detailed investigation of the relationship between exercise-induced improvements in cutaneous microvascular reactivity and ulcer healing), the short-term follow-up (which precluded investigation of cutaneous microvascular adaptations occurring after the exercise programme), and a lack of data exploring the mechanisms underpinning the observed differences and changes in microvascular responsiveness to ACh and SNP. Regarding the latter, further research is warranted to determine the extent to which vascular structural changes in venous disease influence the non-invasive estimates of microvascular function and their clinical significance. An additional limitation was that a lower-extremity venous duplex evaluation was not conducted at baseline to characterise each participant’s venous insufficiency. Nevertheless, the results are representative of what might be expected in a mixed outpatient population.

In summary, we observed an impairment of cutaneous microvascular reactivity in the gaiter area of legs with venous ulcers that was abolished following a 12-week programme of aerobic and resistance exercise. Although the precise mechanisms of these vascular adaptations are unclear, it is possible that exercise training-induced improvements in lower-limb cutaneous microvascular reactivity may facilitate the healing of venous leg ulcers. An appropriately powered trial is needed to clarify the effects of adjunctive exercise training on the healing rates of venous ulcers and the role of different microvascular adaptations in the healing process.

## Abbreviations

ACh: Acetylcholine
ANCOVA: Analysis of covariance
AU: Arbitrary units
CI: Confidence interval
CVC: Cutaneous vascular conductance
SkBF: Skin blood flow
SD: Standard deviation
SNP: Sodium nitroprusside

## References

[CR1] Agale SV (2013) Chronic leg ulcers: epidemiology, aetiopathogenesis, and management. Ulcers. https://doi.org/10.1155/2013/413604

[CR2] Ardron ME, Helme RD, McKernan S (1991). Microvascular skin responses in elderly people with varicose leg ulcers. Age Ageing.

[CR3] Barwell JR, Taylor M, Deacon J, Davies C, Whyman MR, Poskitt KR (2001). Ankle motility is a risk factor for healing of chronic venous leg ulcers. Phlebology.

[CR4] Berghoff M, Kathpal M, Kilo S, Hilz MJ, Freeman R (2002). Vascular and neural mechanisms of ACh-mediated vasodilation in the forearm cutaneous microcirculation. J Appl Physiol.

[CR5] Black MA, Green DJ, Cable NT (2008). Exercise prevents age-related decline in nitric-oxide-mediated vasodilator function in cutaneous microvessels. J Physiol.

[CR6] Borg GA (1982). Psychophysical bases of perceived exertion. Med Sci Sports Exerc.

[CR7] Brunt VE, Fujii N, Minson CT (2015). Endothelial-derived hyperpolarization contributes to acetylcholine-mediated vasodilation in human skin in a dose-dependent manner. J Appl Physiol.

[CR8] Droog EJ, Henricson J, Nilsson GE, Sjöberg F (2004). A protocol for iontophoresis of acetylcholine and sodium nitroprusside that minimises nonspecific vasodilatory effects. Microvasc Res.

[CR9] Eberhardt RT, Raffetto JD (2014). Chronic venous insufficiency. Circulation.

[CR10] Eze AR, Comerota AJ, Cisek PL, Holland BS, Kerr RP, Veeramasuneni R, Comerota AJ (1996). Intermittent calf and foot compression increases lower extremity blood flow. Am J Surg.

[CR11] Foster W, Carruthers D, Lip GY, Blann AD (2010). Inflammation and microvascular and macrovascular endothelial dysfunction in rheumatoid arthritis: effect of treatment. J Rheumatol.

[CR12] Fujii N, Reinke MC, Brunt VE, Minson CT (2013). Impaired acetylcholine-induced cutaneous vasodilation in young smokers: roles of nitric oxide and prostanoids. Am J Physiol Heart Circ Physiol.

[CR13] Galarraga B, Khan F, Kumar P, Pullar T, Belch JJ (2008). C-reactive protein: the underlying cause of microvascular dysfunction in rheumatoid arthritis. Rheumatology.

[CR14] Gardner-Medwin JM, Taylor JY, Macdonald IA, Powell RJ (1997). An investigation into variability in microvascular skin blood flow and the responses to transdermal delivery of acetylcholine at different sites in the forearm and hand. Br J Clin Pharmacol.

[CR15] Graham ID, Harrison MB, Nelson EA, Lorimer K, Fisher A (2003). Prevalence of lower-limb ulceration: a systematic review of prevalence studies. Adv Skin Wound Care.

[CR16] Green DJ, Carter HH, Fitzsimons MG, Cable NT, Thijssen DH, Naylor LH (2010). Obligatory role of hyperaemia and shear stress in microvascular adaptation to repeated heating in humans. J Physiol.

[CR17] Hambrecht R, Adams V, Erbs S, Linke A, Kränkel N, Shu Y, Baither Y, Gielen S, Thiele H, Gummert JF (2003). Regular physical activity improves endothelial function in patients with coronary artery disease by increasing phosphorylation of endothelial nitric oxide synthase. Circulation.

[CR18] Hodges GJ, Sharp L, Stephenson C, Patwala AY, George KP, Goldspink DF, Cable NT (2010). The effect of 48weeks of aerobic exercise training on cutaneous vasodilator function in post-menopausal females. Eur J Appl Physiol.

[CR19] Holowatz LA, Thompson CS, Minson CT, Kenney WL (2005). Mechanisms of acetylcholine-mediated vasodilatation in young and aged human skin. J Physiol.

[CR20] Iabichella ML, Melillo E, Mosti G (2006). A review of microvascular measurements in wound healing. Int J Low Extrem Wounds.

[CR21] Jull A, Parag V, Walker N, Maddison R, Kerse N, Johns T (2009). The PREPARE pilot RCT of home-based progressive resistance exercises for venous leg ulcers. J Wound Care.

[CR22] Jünger M, Steins A, Hahn M, Häfner HM (2000). Microcirculatory dysfunction in chronic venous insufficiency (CVI). Microcirculation.

[CR23] Kan YM, Delis KT (2001). Hemodynamic effects of supervised calf muscle exercise in patients with venous leg ulceration: a prospective controlled study. Arch Surg.

[CR24] Kellogg DL, Zhao JL, Coey U, Green JV (2005). Acetylcholine-induced vasodilation is mediated by nitric oxide and prostaglandins in human skin. J Appl Physiol.

[CR25] Klonizakis M, Yeung JM, Nash JR, Lingam K, Manning G, Donnelly R (2003). Effects of posture and venous insufficiency on endothelial-dependent and -independent cutaneous vasodilation in the perimalleolar region. Eur J Vasc Endovasc Surg.

[CR26] Klonizakis M, Tew GA, Michaels JA, Saxton JM (2009). Exercise training improves cutaneous microvascular endothelial function in post-surgical varicose vein patients. Microvasc Res.

[CR27] Klonizakis M, Tew GA, Michaels JA, Saxton JM (2010). Effects of upper-limb exercise on lower-limb cutaneous microvascular function in post-surgical varicose-vein patients. Eur J Appl Physiol.

[CR28] Klonizakis M, Tew GA, Gumber A, Crank H, King B, Middleton G, Michaels JA (2017) Supervised exercise training as an adjunct therapy for venous leg ulcers: a randomised controlled feasibility trial. Br J Dermatol. https://doi.org/10.1111/bjd.16089 10.1111/bjd.16089 PMC6001633 29077990

[CR29] Lanting SM, Johnson NA, Baker MK, Caterson ID, Chuter VH (2017). The effect of exercise training on cutaneous microvascular reactivity: a systematic review and meta-analysis. J Sci Med Sport.

[CR30] Margolis DJ, Berlin JA, Strom BL (1999). Risk factors associated with the failure of a venous leg ulcer to heal. Arch Dermatol.

[CR31] Meissner MH, Moneta G, Burnand K, Gloviczki P, Lohr JM, Lurie F, Mattos MA, McLafferty RB, Mozes G, Rutherford RB (2007). The hemodynamics and diagnosis of venous disease. J Vasc Surg.

[CR32] Middlebrooke AR, Elston LM, Macleod KM, Mawson DM, Ball CI, Shore AC, Tooke JE (2006). Six months of aerobic exercise does not improve microvascular function in type 2 diabetes mellitus. Diabetologia.

[CR33] Morris SJ, Shore AC (1996). Skin blood flow responses to the iontophoresis of acetylcholine and sodium nitroprusside in man: possible mechanisms. J Physiol.

[CR34] Nielsen HV (1983). Arterial pressure-blood flow relations during limb elevation in man. Acta Physiol Scand.

[CR35] O’Brien J, Edwards H, Stewart I, Gibbs H (2013). A home-based progressive resistance exercise programme for patients with venous leg ulcers: a feasibility study. Int Wound J.

[CR36] Qiu J, Zheng Y, Hu J, Liao D, Gregersen H, Deng X, Fan Y, Wang G (2013). Biomechanical regulation of vascular smooth muscle cell functions: from in vitro to in vivo understanding. J R Soc Interface.

[CR37] Raffetto JD (2016). Pathophysiology of wound healing and alterations in venous leg ulcers-review. Phlebology.

[CR38] Scottish Intercollegiate Guidelines Network (2010) Guideline No. 120: Management of chronic venous leg ulcers. http://www.sign.ac.uk/guidelines/fulltext/120/. Accessed 30 Aug 2017

[CR39] Simka M (2007). Calf muscle pump impairment and delayed healing of venous leg ulcers: air plethysmographic findings. J Dermatol.

[CR40] Simmons GH, Wong BJ, Holowatz LA, Kenney WL (2011). Changes in the control of skin blood flow with exercise training: where do cutaneous vascular adaptations fit in?. Exp Physiol.

[CR41] Tew GA, Klonizakis M, Saxton JM (2010). Effects of ageing and fitness on skin-microvessel vasodilator function in humans. Eur J Appl Physiol.

[CR42] Tew GA, Michaels J, Crank H, Middleton G, Gumber A, Klonizakis M (2015). Supervised exercise training as an adjunctive therapy for venous leg ulcers: study protocol for a randomised controlled trial. Trials.

[CR43] Turner J, Belch JJ, Khan F (2008). Current concepts in assessment of microvascular endothelial function using laser Doppler imaging and iontophoresis. Trends Cardiovasc Med.

[CR44] Vickers AJ, Altman DG (2001). Analysing controlled trials with baseline and follow up measurements. BMJ.

[CR45] Wang JS (2005). Effects of exercise training and detraining on cutaneous microvascular function in man: the regulatory role of endothelium-dependent dilation in skin vasculature. Eur J Appl Physiol.

[CR46] Yim E, Kirsner RS, Gailey RS, Mandel DW, Chen SC, Tomic-Canic M (2015). Effect of physical therapy on wound healing and quality of life in patients with venous leg ulcers: a systematic review. JAMA Dermatol.

